# Harnessing AI-driven approaches for detecting metabolic dysfunction-associated steatotic liver disease, assessing fibrosis, and stratifying hepatocellular carcinoma risk: a scoping review

**DOI:** 10.3389/fonc.2026.1803957

**Published:** 2026-07-01

**Authors:** Anvitha Nagaraj Sharma, Hima Bhagavatula, Michael T. Mapundu, Emile R. Chimusa

**Affiliations:** 1 Bioinformatic & Multi-Omics Data Science Group, Faculty of Sciences and Environment, Northumbria University, Newcastle, United Kingdom; 2 AI and Data-Driven Department, Omics Data Solutions Limited, Dublin, Ireland

**Keywords:** artificial intelligence, hepatocellular carcinoma, machine learning, metabolic dysfunction associated steatotic liver disease, multimodal, multi-omics, non-alcoholic fatty liver disease, polygenic risk score

## Abstract

**Introduction:**

Non-alcoholic fatty liver disease (NAFLD) is the most prevalent chronic liver disorder worldwide and a major risk factor for hepatocellular carcinoma (HCC). Its rising prevalence and progression to HCC present major clinical and public health challenges. Current diagnostic and prognostic tools lack accuracy in predicting disease progression and early HCC risk. Emerging approaches, including artificial intelligence (AI), machine learning (ML), and polygenic risk scores (PRS) offer promising opportunities and improvements in non-invasive diagnosis, risk stratification, and precision medicine.

**Methods:**

We conducted a scoping review synthesising evidence on the application of PRS and AI/ML models for predicting and detecting MASLD, assessing fibrosis, and stratifying risk of HCC among individuals with MASLD/NAFLD, with a particular focus on European and Asian populations. The review was performed in accordance with PRISMA 2020 guidelines and aimed to map the development trajectory and knowledge structure of predictive approaches for MASLD/NAFLD–HCC risk stratification, while identifying key translational challenges and opportunities.

**Results:**

Evidence from the included studies indicates a shift from isolated methodological innovation towards integrated, explainable, and clinically validated multimodal models, supported by transparent AI/ML systems aligned with regulatory frameworks. There is also a critical need for large, multi-centre validation studies and interdisciplinary collaboration among clinicians, data scientists, and policymakers to enable scalable and equitable implementation. Furthermore, progress depends on moving from theoretical promise to clinical reality through prospective trials that assess real-world effectiveness and adoption pathways.

**Conclusion:**

This review highlights advances in AI and PRS for HCC risk prediction in MASLD/NAFLD, demonstrating that multimodal models outperform traditional approaches. AI-driven methods show strong potential for MASLD detection, fibrosis assessment, and HCC risk stratification, with implications for improved clinical outcomes. However, translation into practice is limited by poor genetic integration, lack of validation, population bias, and limited explainability. Further validation, standardization, and clinical integration are required for widespread adoption and effective personalised surveillance.

## 1 Introduction

Non-alcoholic fatty liver disease (NAFLD), recently redefined as metabolic dysfunction-associated steatotic liver disease (MASLD), is characterised by excessive hepatic fat accumulation in the absence of significant alcohol intake or other secondary causes (1, 2). It is now the most common chronic liver disease worldwide, affecting around 25% of the adult population, with particularly high prevalence in Western and Asia-Pacific regions (3–8). MASLD/NAFLD is closely associated to metabolic syndrome, including obesity, insulin resistance, and type 2 diabetes mellitus, and encompasses a spectrum ranging from simple steatosis to non-alcoholic steatohepatitis (NASH), fibrosis, cirrhosis, and hepatocellular carcinoma (HCC) (1, 9–13).

The global burden of MASLD/NAFLD is rising rapidly, driven by increasing metabolic risk factors, and is projected to become a leading indication for liver transplantation (14, 15). Prevalence varies geographically, ranging from 13-18% in Africa to up to 48% in Europe (3, 16, 17). Importantly, MASLD/NAFLD can progress to HCC through both cirrhotic and non-cirrhotic pathways, with up to 25% of cases occurring without cirrhosis, limiting current surveillance strategies (18, 19). HCC remains a major cause of cancer mortality, accounting for over 830,000 deaths annually (20–23). In patients with NASH, annual HCC incidence ranges from 0.5 to 2.6% (24).

Disease progression is driven by metabolic and inflammatory mechanisms, including lipotoxicity, oxidative stress, and genomic instability (25–29), alongside cytokine-mediated fibrosis and adipokine dysregulation (30, 31). Genetic susceptibility further modifies risk, with variants in *PNPLA3, TM6SF2, MBOAT7, HSD17B13*, and *GCKR* consistently associated with disease severity and HCC development (32–42). Polygenic risk scores (PRS) aggregate these effects and show predictive value, although genetic risk alone remains insufficient due to substantial disease heterogeneity (15, 43–46).

Artificial intelligence (AI), including machine learning (ML) and deep learning (DL), enables modelling of complex, nonlinear interactions across clinical, imaging, and multi-omics data (47–49). These approaches have improved disease classification, lesion detection, and risk prediction in MASLD/NAFLD and HCC (50–54), while initiatives such as LITMUS support multimodal integration (55). Importantly, AI can enhance PRS by incorporating gene–environment interactions and diverse data sources (56, 57).

Despite these advances, research remains fragmented, often focusing on single modalities such as imaging or genetics (58–63). There is a lack of integrated frameworks combining AI/ML, PRS, and multi-omics data for MASLD/NAFLD-associated HCC prediction, particularly across European and Asian populations. Given the rising global burden, including projections exceeding 314 million cases in China and high prevalence in Europe, there is an urgent need for scalable, population-specific risk stratification strategies (16, 64, 65). This scoping review therefore synthesizes recent evidence (2020–2025) on AI/ML and PRS-based approaches on AI/ML and PRS-based approaches for MASLD early detection, fibrosis assessment, and HCC risk stratification, aiming to identify methodological gaps and support the development of clinically actionable precision medicine strategies.

## 2 Methodology

### 2.1 Defining the research question

This scoping review was conducted in accordance with the Preferred Reporting Items for Systematic Reviews and Meta-Analyses (PRISMA) 2020 guidelines (66) to ensure transparency, reproducibility, and methodological rigor. The Population, Intervention, Comparator, Outcome, Study Design (PICOS) framework was used to define the research question and guide study selection, focusing on the application of AI/ML and PRS for MASLD early detection, fibrosis assessment, and HCC risk stratification in individuals with NAFLD. The review emphasized studies published between 2020 and 2025 in European and Asian populations.

### 2.2 Eligibility criteria

Rigorous inclusion and exclusion criteria were applied to identify high-quality studies relevant to the objectives of this review. Given the rapid evolution of AI/ML, and PRS research, only powered, peer-reviewed studies published within the past five years were considered eligible. This approach ensures that the review captures the most recent methodological advances, innovations, and translational applications pertinent to NAFLD-associated HCC.

Studies were included if they:

1. Were written in English and involved human participants of European or Asian ancestry.
2. Focused on NAFLD, NASH, cirrhosis, or NAFLD-associated HCC.
3. Applied PRS and/or AI/ML approaches for early detection, diagnosis, or risk stratification; and
4. Reported relevant outcomes, including risk associations, predictive performance metrics, early detections, and AI/ML model evaluations.
5. Were peer reviewed original research articles published between 2020 and 2025.
6. Were powered observational studies (cohort, case-control, cross-sectional), GWAS, longitudinal studies, PRS validations.

Studies were excluded if they were non-English publications, conference abstracts, reviews, meta-analyses, editorials, case reports, and grey literature. Animal studies or clinical studies lacking PRS or AI/ML components were excluded. Populations not of European or Asian ancestry and studies not focused on NAFLD-related conditions were omitted. Additionally, studies relying solely on clinical, radiological, or histological diagnosis without PRS or AI/ML approaches, or those not reporting relevant outcomes, were excluded.

### 2.3 Literature search strategy

A systematic literature search was conducted across two English-language databases, PubMed and Web of Science. These databases were selected for their comprehensive coverage of biomedical and clinical research relevant to NAFLD, HCC, genetics, and AI/ML methodologies. Preliminary searches were also performed in Scopus, Embase, and Google Scholar; however, PubMed and Web of Science yielded the most relevant and methodologically robust and high-quality records.

Search strategies were developed using a combination of Medical Subject Headings (MeSH) and free-text terms. The primary search string used across all databases was “*machine learning for early detection of NAFLD and its progression to HCC*”. This core query was subsequently refined and expanded using Boolean operators and alternative keywords to maximize the retrieval of relevant studies. To ensure comprehensive coverage and account for evolving nomenclature, synonymous terms were incorporated during search refinement, including the recently adopted term “metabolic dysfunction–associated steatotic liver disease (MASLD)” as a replacement for NAFLD. Both terms were included to capture the full breadth of relevant literature across transitional terminology.

The complete Boolean logic applied was *(“Non-alcoholic fatty liver disease” OR “NAFLD” OR “NASH”*) AND *(“hepatocellular carcinoma”* OR “*HCC*” OR “*liver cancer*”).

AND (“*polygenic risk score*” OR “*PRS*” OR “*genetic risk*” OR “*GWAS*”).

AND (“*machine learning*” OR “*artificial intelligence*” OR “*deep learning*” OR “*predictive model*” OR “*AI-driven diagnostic*” OR “*early detection*” OR “*risk stratification*”).

AND (“*Europe” OR “European population*” OR “*Asia*” OR “*Asian population*”).

Search strategies were iteratively refined through pilot searches, during which alternative keywords were tested, and Boolean operators were adjusted to optimize sensitivity and specificity. Reference lists of articles included were also screened to identify additional relevant studies while excluding grey literature. Synonymous terms were incorporated where appropriate, and MeSH mapping was verified in PubMed. Search results were subsequently compared across the databases to ensure completeness prior to final implementation.

### 2.4 Study selection and de-duplication

**Figure 1:**
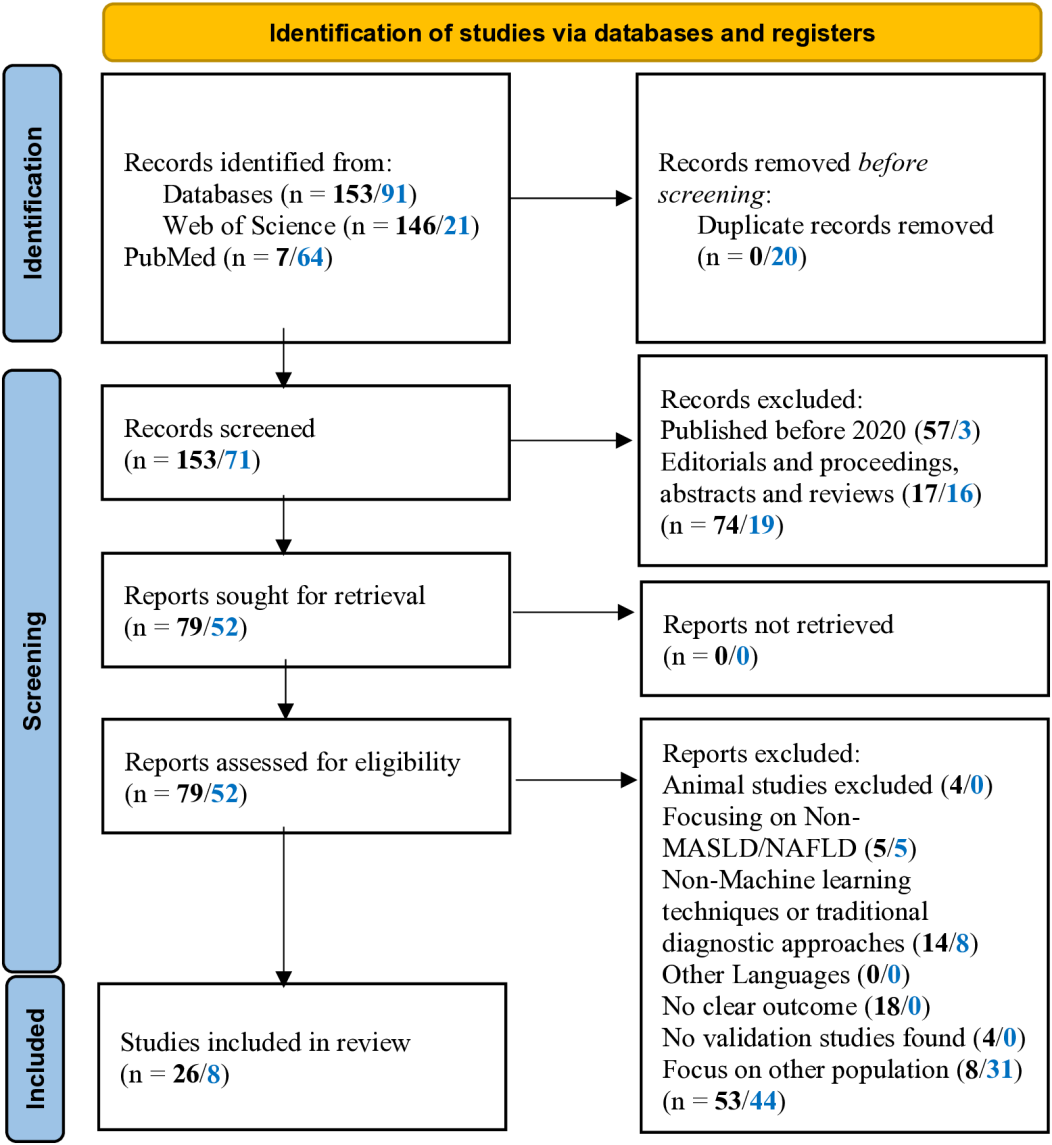
A PRISMA 2020 flowchart depicting the study selection process, showing record counts at each stage and reasons for exclusion. Blue and bold numbers are from European and Asian populations, respectively. This figure illustrates the study selection process, including identification, screening, eligibility assessment, and final inclusion of studies according to PRISMA 2020 guidelines.

All records retrieved from the databases were exported into EndNote 21 for de-duplication. Both automated and manual de-duplication procedures were applied by matching titles, authors, digital object identifiers (DOIs), and journal information to ensure each study was represented only once. Titles and abstracts were then independently screened against the predefined eligibility criteria. Full texts of potentially eligible studies were subsequently assessed for inclusion. Manual screening was used to confirm that all included studies met the inclusion criteria, and to ensure the appropriate exclusion of review articles, editorials, and other non-eligible publication types. Notably, most included studies focused on MASLD detection and fibrosis assessment, while only a limited number directly addressed HCC prediction. **Figure 1** shows a flow chart of the study selection process.

### 2.5 Data extraction, quality assessment, risk of bias and statistical analyses

A structured approach was utilized to collect relevant information from the studies included. The extracted data included; (1) study details, including author names, year of publication, journal, population ancestry, country of study, and sample size; (2) stage of disease progression; (3) genomic methodologies applied; (4) AI/ML algorithms employed; (5) study outcomes and (6) sources of data and (6) reported limitations. Study quality was assessed using the Newcastle-Ottawa Scale (NOS) for non-randomised observational and prognostic studies (67–71). Quality Assessment of Diagnostic Accuracy Studies 2 (QUADAS-2) reported in Matuszewska et al. (72–75) was used for diagnostic accuracy studies (76, 77). This dual quality assessment approach was adopted to account for methodological heterogeneity across the included studies. Descriptive statistical analyses were conducted to summarise key study characteristics and to examine the frequency and distribution of AI/ML algorithms applied across the included studies. To enhance transparency, the quality assessments were explicitly considered in the interpretation of the results, with particular attention to potential sources of bias, sample representativeness, and reporting standards, and how these factors may have influenced reported model predictive performance.

## 3 Results

**Figure 2:**
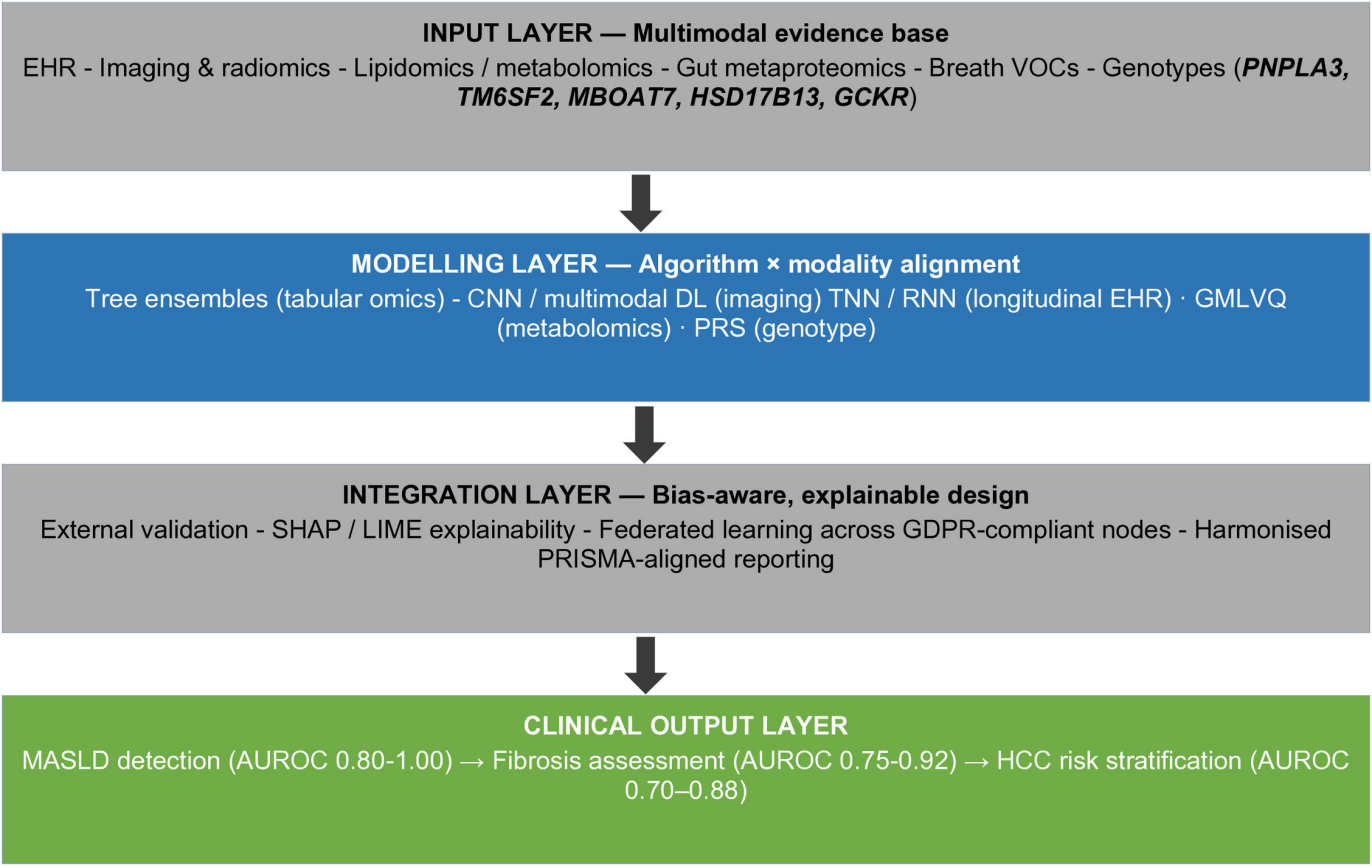
Synthesis pathway from data inputs to clinical outputs for included studies.

A total of 244 records were identified from PubMed and Web of Science. These were made up of 153 studies from Asian populations and 91 from European populations. Following automated and manual de-duplication, titles and abstracts were screened against the predefined eligibility criteria. Full-text assessment resulted in the inclusion of 34 studies, of which 26 (76%) were conducted in Asian populations and 8 (24%) in European populations, published between 2020 and 2025. All included studies investigated the application of PRS, AI, and/or ML approaches for NAFLD-related outcomes, including early detection, diagnosis, risk stratification, or progression to HCC. Cohort sizes ranged from 80 to 36,490 participants, reflecting substantial heterogeneity in study scale. The study selection process is depicted in **Figure 1** (all records highlighted in blue and black are from European and Asian populations respectively). Additionally, the populations, outcomes, and main clinical focus of the included studies are summarised in **Supplementary Table 1**. A four-layer schematic diagram in **Figure 2** synthesises how the included studies move from heterogeneous inputs (clinical, imaging, omics, genotype) through algorithm-modality alignment and bias-aware integration, to clinically actionable outputs across the MASLD, fibrosis and HCC continuum. AUROC ranges are summary values from **Table 1**.

**Table 1:** Summary of AI/ML algorithms: frequency, application context, and performance.

Algorithm	Frequency	Proportion	Primary use context	Typical AUROC
Random Forest (RF)	22	65%	Omics, clinical features, screening	0.82 - 0.99
Support Vector Machine (SVM)	16	47%	Biomarker pipelines, screening	0.80 - 0.94
XGBoost/Gradient Boosting	15	44%	EHR, multi-centre studies	0.83 - 0.94
LASSO Regression	10	29%	Feature selection in omics studies	0.89 - 0.997
Logistic Regression	9	26%	Baseline comparator, clinical scoring	0.73 - 0.93
Deep Learning (DL/TNN)	6	18%	Longitudinal EHR, multimodal	0.85 - 0.93
KNN	5	15%	Screening, clinical phenotyping	0.80 - 0.91
LightGBM	4	12%	Large-scale screening	0.81 - 0.92
Transformer/TNN	2	6%	Longitudinal mortality prediction	0.70 - 0.93

RF, Random Forest; SVM, Support Vector Machine; LR, Logistic Regression; DL, Deep Learning; TNN, Tensor Neural Networks; EHR, Electronic Health Record.

### 3.1 Study quality and risk of bias

**Figure 3:**
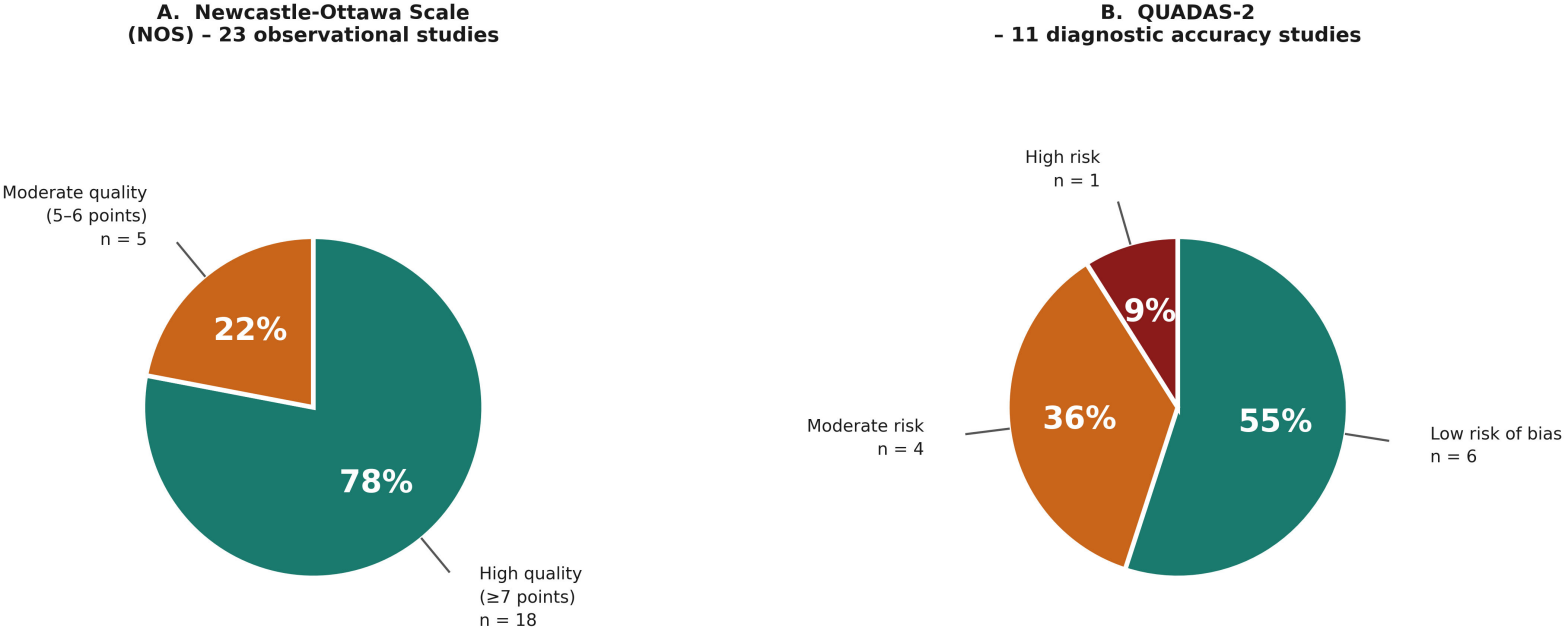
Summary of study quality assessments. **(A)** NOS ratings: 78% (n = 18) achieved high quality (>=7 points); 22% (n = 5) were moderate quality (5–6 points). **(B)** QUADS-2 risk of bias: 55% (n=6) low risk; 36% (n=4) moderate risk; 9% (n=1) high risk.

Study quality was evaluated using the Newcastle-Ottawa Scale (NOS) for 23 non-randomised observational studies and QUADAS-2 for 11 diagnostic accuracy studies. Results are summarised in **Figure 3**.

Using the NOS, a total of 23 non-randomised observational or prognostic studies were evaluated. Of these, 18 (78%) were rated as high quality (≥7 points), while five (22%) were classified as moderate quality (5–6 points); no study scored below the threshold indicative of poor quality. High quality ratings were observed in large, multi-centre, or population-based cohorts with clear outcome definitions, comprehensive confounder adjustment, and adequate follow-up. Single-centre studies more frequently lacked detailed follow-up or sufficient confounder control, yielding moderate quality scores. For diagnostic accuracy studies assessed using QUADAS-2, 6 (55%) demonstrated overall low risk of bias, 4 (36%) were rated moderate risk, and one (9%) was classified as high risk. Principal sources of bias included insufficient reporting of participant flow and unclear timing between index tests and reference standards.

Across both tools, higher methodological quality was generally associated with more conservative yet reproducible performance estimates, whereas moderate-quality studies more frequently reported elevated internal validation metrics without external replication. These findings underscore the importance of rigorous study design, transparent reporting, and external validation in the development and evaluation of predictive models for MASLD detection, fibrosis assessment, and HCC risk stratification.

### 3.2 Distribution of studies by clinical focus

**Figure 4:**
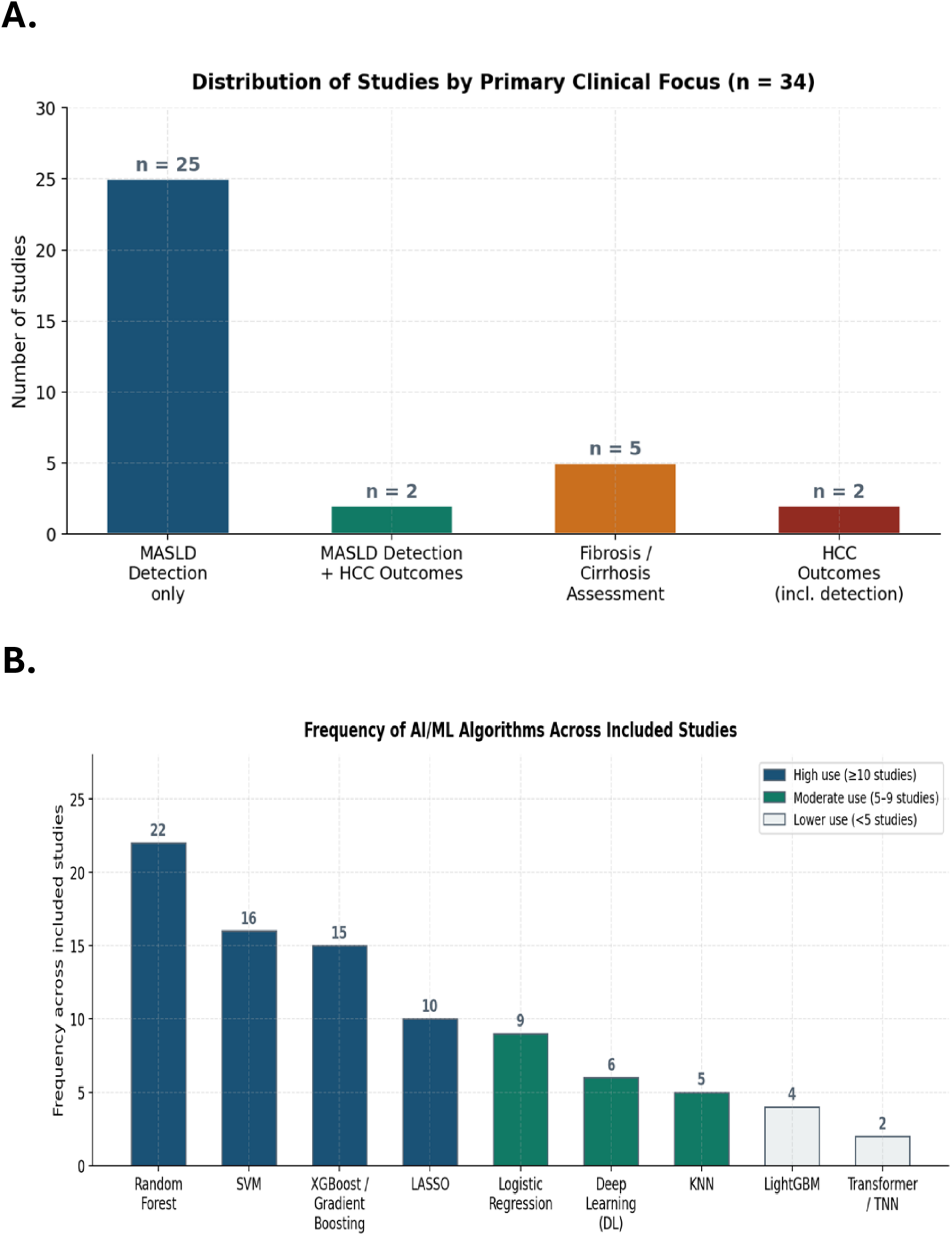
**(A)** Distribution of included studies by primary clinical focus. MASLD detection constituted 74% of all studies (n = 25), reflecting the predominant research emphasis on early-stage disease identification. Fibrosis/cirrhosis assessment comprised 15% (n = 5), and HCC-related outcomes 12% (n = 4 across two subcategories). **(B)** Frequency of AI/ML algorithms across included studies (n = 34). Random Forest was the most widely applied (22 studies; 65%), followed by SVM (16; 47%) and XGBoost/Gradient Boosting (15; 44%). Deep learning approaches were used in 6 studies (18%). Colour coding indicates high use (blue, >=10), moderate use (teal, 5-9), and lower use (grey, <5).

Included studies were categorised into four primary clinical focus groups. As shown in **Figure 4A** and summarised in **Table 2**, MASLD detection was the predominant focus (n = 25; 74%), followed by fibrosis or cirrhosis assessment (n = 5; 15%), and HCC-related outcomes either alone (n = 2; 6%) or combined with MASLD detection (n = 2; 6%). This distribution reflects the current clinical emphasis on early disease identification, with fewer studies addressing the more complex downstream endpoints of advanced fibrosis and HCC risk.

**Table 2:** Structured synthesis of included studies by clinical focus category.

Clinical focus	Studies (n)	Sample size range	Top algorithm(s)	AUROC range
MASLD Detection only	25 (74%)	80 - 36,490	RF, XGBoost, LightGBM	0.75 - 1.00
Fibrosis/Cirrhosis Assessment	5 (15%)	80 - 2,472	RF, LR, SVM	0.73 - 0.99
HCC-related Outcomes	2 (6%)	80 - 249	RF, Gradient Boosting	0.80 - 0.997
MASLD Detection + HCC	2 (6%)	14,439 - 14,913	SVM, RF	0.85 - 0.92

RF, Random Forest; LR, Logistic Regression; SVM, Support Vector Machine; AUROC, Area Under the Receiver Operating Characteristic curve.

MASLD detection studies not only dominate numerically but also encompass the widest diversity of algorithmic approaches and data modalities, including genomic bioinformatics, imaging radiomics, and breath-based biomarkers. Fibrosis and HCC studies, while fewer, often achieved comparable or superior predictive performance, particularly in omics-driven frameworks, suggesting that focused, hypothesis-driven designs can compensate for smaller sample sizes in terms of discriminatory performance, albeit with associated limitations in generalisability.

Tree-based ensemble methods, particularly Random Forest and Gradient Boosting, were the most frequently used algorithms due to their ability to handle high-dimensional data. Support Vector Machines were commonly applied in biomarker-based pipelines, while deep learning approaches were increasingly employed in multimodal and longitudinal frameworks. Feature selection methods such as LASSO regression, SVM-RFE, and Boruta were often combined with ensemble models in omics-based investigations.

### 3.3 AI/ML algorithm landscape

Across the 34 included studies, a broad range of AI/ML algorithms were applied. Random Forest (RF), Support Vector Machine (SVM), and XGBoost/Gradient Boosting emerged as the three most frequently used methods (**Figure 4B**, **Table 1**). Tree-based ensemble methods predominated in high-dimensional feature-rich datasets, including serum lipidomics (73), fecal metaproteomics (75), and large-scale clinical registries (69, 70). LASSO regression appeared predominantly as a feature selection tool within ensemble pipelines in omics-focused studies rather than as a standalone classifier. Deep learning architectures, including Transformer neural networks and multimodal frameworks, were applied in six studies (18%), typically to longitudinal EHR data (68) or multimodal clinical imaging phenotypic datasets (78). LightGBM was deployed in large-scale population screening studies, demonstrating competitive performance with reduced computational cost.

A key observation is that algorithm selection was broadly aligned with data structure: ensemble tree methods dominated in feature-rich omics and clinical datasets, while neural network architectures were reserved for longitudinal and multimodal contexts. This alignment was associated with stronger external validation performance, suggesting that effective MASLD/HCC risk modelling depends less on identifying a universally optimal algorithm and more on matching modelling strategy to the inherent structure and biological relevance of the underlying data.

### 3.4 Data modalities and their relationship to model performance

**Figure 5:**
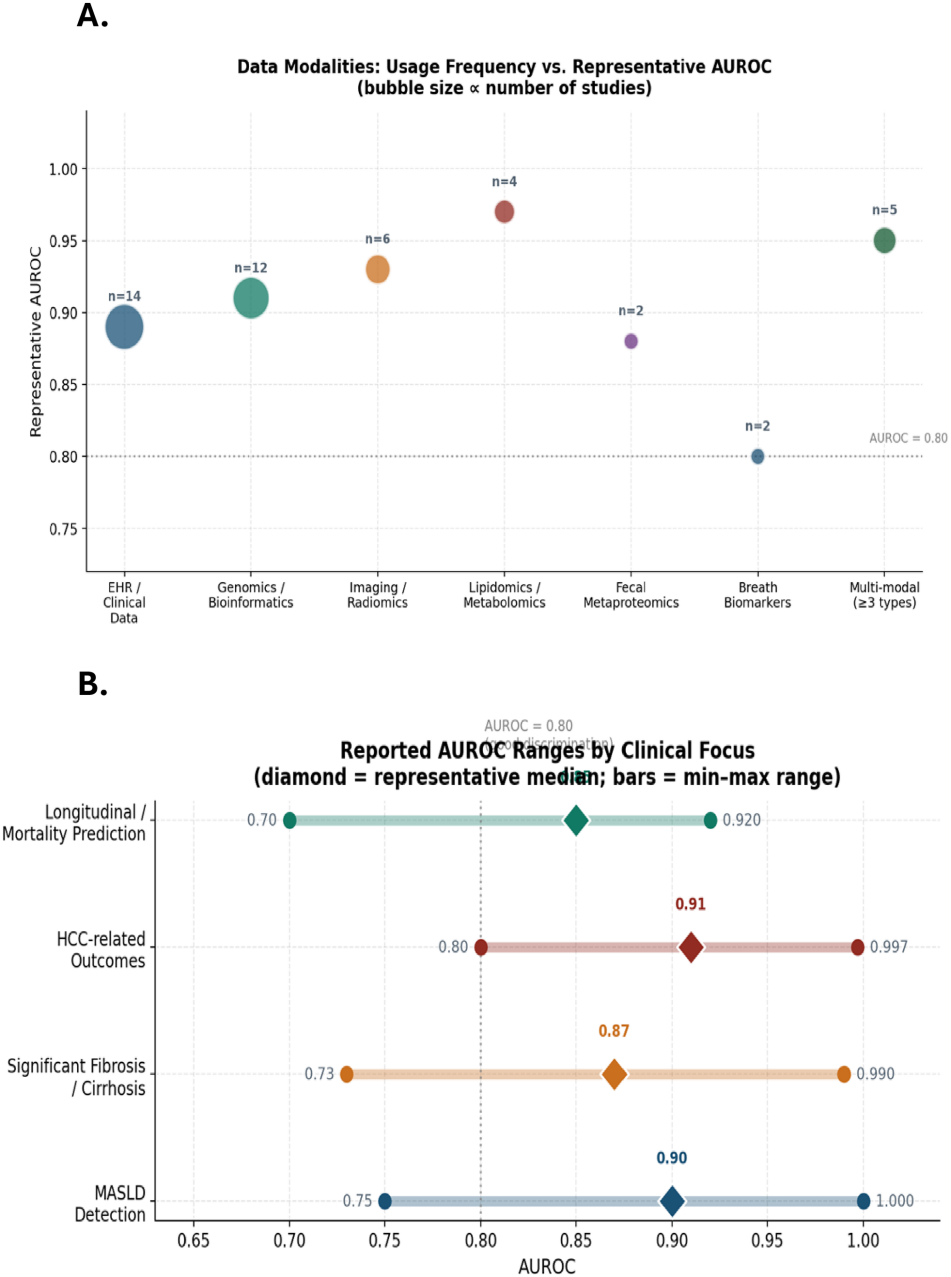
**(A)** Data modality frequency versus representative AUROC. Bubble size is proportional to the number of studies using each modality. EHR/clinical data and genomics/bioinformatics were the most frequently applied. Lipidomics/metabolomics achieved the highest representative AUROCs (up to 0.997), though typically in smaller single-centre cohorts. Novel modalities (breath biomarkers, fecal metaproteomics) were emerging and exploratory. **(B)** Reported AUROC ranges by clinical focus category. Diamonds denote representative median AUROC; horizontal bars represent min-max observed range. MASLD detection showed the widest range (0.75-1.00), reflecting heterogeneity in modality and cohort size. Fibrosis/cirrhosis and HCC studies achieved comparable or superior median AUROCs, while longitudinal mortality prediction demonstrated the widest downward spread (AUROC reaching 0.70 in external validation).

Included studies leveraged a diverse range of data modalities. **Figure 5A** maps each modality by frequency of use (bubble size) against representative AUROC, providing a visual synthesis of both breadth of application and diagnostic yield. EHR and clinical phenotypic data were the most used (n = 14 studies), reflecting their availability and compatibility with large-scale population cohorts (71). Genomic and bioinformatics approaches were applied in 12 studies, predominantly for MASLD detection and mechanistic biomarker discovery, including ferroptosis-related gene identification (79). Imaging and radiomics approaches (n = 6 studies) achieved strong AUROC in structured validation settings (e.g., AUC 0.885 external validation; 80).

Lipidomic and metabolomic profiling (n = 4 studies) consistently yielded the highest AUROC values, including near-perfect discrimination (AUC 0.997) in a four-gene diagnostic model (81) and a GMLVQ-based urinary steroid metabolome classifier (74). However, these findings were derived from smaller, single-centre cohorts with limited generalisability. Novel non-invasive modalities, VOC breath testing (82) and lactulose breath test analysis (83), represent clinically attractive emerging approaches, particularly for resource-limited settings. Multimodal integration (n = 5 studies) was consistently associated with higher predictive accuracy; DeepFLD (78) achieved superior discrimination by combining laboratory data, imaging, clinical parameters, and facial photographs. This performance gradient, from omics and multimodal approaches to imaging and finally EHR/clinical-only models, underscores the need for integrated frameworks that combine the precision of omics with the scalability of EHR-based systems, an objective that has not yet been systematically achieved in the current literature.

### 3.5 Predictive performance across clinical focus categories

Overall, model performance varied across studies, with AUROC values ranging from 0.70 in external mortality prediction to 1.00 in highly controlled metabolomic classification tasks. **Figure 5B** summarises AUROC distributions across the four primary clinical focus categories. The dashed vertical line at AUROC = 0.80 denotes the accepted threshold for good discriminatory performance in clinical prediction models.

MASLD detection studies demonstrated the widest AUROC range (0.75-1.00), reflecting substantial diversity in algorithmic approaches, cohort sizes, and data modalities. Most studies reported strong discriminatory performance (AUROC >= 0.80) during internal validation. Bioinformatics driven and omics-based models tended toward higher AUROCs, while large population-based screening studies, constrained by noisier, less curated data, reported more moderate but clinically meaningful values (e.g., SVM AUROC 0.85; 69). Fibrosis and cirrhosis assessment models reported AUROCs between 0.73 and 0.99, with several studies prioritising high negative predictive value (NPV) as the primary performance metric, reflecting their intended clinical utility as rule-out tools to reduce unnecessary biopsies (NPV 0.947, (84) NPV 0.958 for cirrhosis, (85)).

HCC-related outcome studies, though fewer in number, achieved strong AUROC values (0.80-0.997), with lipidomic-based classifiers demonstrating particularly high discrimination (73, 81, 86). Longitudinal and mortality prediction models employing deep learning on EHR data showed the most performance variability, reaching AUROC 0.92 in internal validation (68) but declining to 0.70 in external mortality prediction, highlighting the persistent challenge of cross-population generalisability. Collectively, performance differences across categories were attributable to three interacting factors: (i) data modality richness, with omics-integrated models consistently outperforming clinical-only models; (ii) validation design, with external validation cohorts yielding lower but more reliable estimates; and (iii) cohort size and representativeness, where smaller single-centre studies reported higher internal metrics without external replication. These observations collectively emphasise that AUROC values in isolation are insufficient benchmarks for clinical translation; external validation, calibration, and net reclassification improvement must also be considered.

### 3.6 Emerging and novel diagnostic approaches

Several studies introduced methodological innovations extending AI-driven diagnostics beyond conventional clinical and imaging pipelines. DeepFLD (78) integrated clinical laboratory data, imaging, questionnaires, and facial photographs into a single multimodal architecture, surpassing clinical-only benchmarks and maintaining acceptable accuracy even when imaging data were unavailable, a finding with direct implications for resource-limited settings. Transformer neural networks applied to longitudinal EHR data (68, 70) demonstrated the feasibility of temporal modelling for mortality prediction and prognostic stratification, representing a conceptual advance beyond cross-sectional risk scoring approaches. Radiomics-based models applied to ultrasound and elastography data (80) achieved strong external validation performance (AUC 0.885), suggesting that structured feature extraction from existing imaging modalities can substantially enhance predictive value without requiring additional investigations.

Explainability tools were incorporated in a subset of studies: SHAP-based feature importance was reported in Yang et al. (87), Tian et al. (88), and Makkena & Natarajan (89), and Yu et al. (90), providing transparent identification of key clinical predictors and enhancing model interpretability for potential clinical adoption. However, most included studies did not incorporate explainability frameworks such as SHAP or LIME, a critical gap given that model interpretability is a prerequisite for regulatory acceptance and clinician trust, particularly for high-stakes decisions in HCC surveillance.

### 3.7 Polygenic risk scores and genomic integration

Despite constituting a central objective of this review, formal PRS-based analyses were explicitly incorporated in only one included study. Atabaki-Pasdar et al. (67) developed a PRS for fatty liver disease within the IMI DIRECT cohort (n = 3,029), integrating genetic variants with clinical and metabolic features in a multi-modal ML framework that achieved AUROC of up to 0.87, demonstrating proof-of-concept for genetics-informed AI/ML risk prediction. The broader literature, however, reflects a critical methodological fragmentation: genomic bioinformatics studies (n = 12) identified novel gene signatures relevant to NAFLD pathogenesis, but these were not integrated into clinically deployable risk stratification frameworks. Established susceptibility variants (*PNPLA3, TM6SF2, MBOAT7, HSD17B13, GCKR*) were rarely incorporated into predictive models, despite consistent evidence for their association with MASLD severity and HCC risk. This separation between genomic discovery and AI/ML-based clinical modelling limits the capacity of existing frameworks to capture gene-environment interactions central to NAFLD pathogenesis and likely leads to underestimation of true disease heterogeneity, particularly for long-term outcomes such as HCC development. Bridging this gap through PRS-augmented multimodal AI frameworks represents one of the most significant unmet methodological needs identified by this review.

### 3.8 Clinical significance and translational implications

The clinical significance of the reviewed AI-driven tools extends beyond their reported AUROC values and has direct implications for how these models could be deployed across different points in the MASLD/NAFLD care pathway. For population-level screening, models built on routine blood test parameters and anthropometric measurements in large cohorts (n > 14,000), particularly LightGBM and RF classifiers, require no specialist imaging infrastructure and are therefore compatible with primary care settings. For fibrosis assessment, high NPV values reported across several ML models suggest a clinically meaningful role in ruling out advanced disease and reducing unnecessary biopsy referrals (91). For HCC surveillance, AI-driven prognostic stratification has the potential to enable earlier detection and more targeted intervention, which are critical determinants of survival in this population. Collectively, these applications suggest that AI/ML frameworks could augment, rather than replace, existing diagnostic pathways, provided that the translational challenges identified in this review are systematically addressed. Realising this clinical potential will require prospective implementation studies and health economic evaluations that move beyond internal validation performance, assessing real-world effectiveness, cost, and adoption pathways across diverse healthcare settings.

## 4 Discussion

Traditional diagnostic approaches for NAFLD, including liver biopsy, imaging, and serum biomarkers, are constrained by invasiveness and limited long-term predictive capacity (92–94). These constraints have motivated growing interest in AI/ML and PRS as tools for precision risk stratification. However, despite their promise, translation into routine clinical practice remains limited. Although PRS development was explored by Atabaki-Pasdar et al. (67), the systematic integration of PRS within AI/ML frameworks for MASLD detection, fibrosis assessment, and HCC risk stratification remains underdeveloped.

This scoping review demonstrates that AI/ML approaches, when applied to multimodal and multi-omics data, offer meaningful advances in early detection, risk stratification, and prognostic evaluation for NAFLD progression to HCC across European and Asian populations. Notably, most included studies concentrated on earlier disease stages, MASLD detection and fibrosis assessment, with comparatively fewer addressing HCC prediction directly, a distribution that reflects both the current state of the field and its most pressing clinical priorities. Despite this breadth of activity, the absence of PRS integration within AI/ML frameworks represents the most consequential methodological gap: genomic discovery and clinical predictive modelling have advanced largely in parallel, limiting the capacity of existing tools to capture the gene–environment interactions that drive long-term disease heterogeneity, particularly HCC development.

Across included studies, AI/ML models were trained on several heterogeneous data, such as EHRs, lipidomics, metabolomics, and fecal metaproteomics. Most studies reported strong discriminatory performance, with AUROC values ≥ 0.80, and peak values ranging from 0.92 to 1.00 in selected biomarker driven contexts. Tree- based ensemble methods were frequently applied to omics, and clinical datasets, while SVMs were commonly used for screening and longitudinal risk modelling using EHR data. Although PRS was not widely integrated into AI/ML models within the included studies, advances in multi-omics analyses across European and Asian cohorts suggest clear feasibility for genetics-informed predictive frameworks. The current fragmentation, where genomic and AI/ML-based clinical models are typically developed in isolation, limiting the ability to gene-environment interactions that are central to NAFLD pathogenesis. From an inferential standpoint, this fragmentation likely leads to underestimation of true disease heterogeneity, particularly for long-term outcomes such as HCC.

A consistent interpretive finding across the included literature is that predictive performance depends less on selecting a single optimal algorithm and more on matching modelling strategy to the structure and biological relevance of the underlying data. Ensemble tree methods excelled in high-dimensional omics and heterogeneous clinical datasets, domain-adapted algorithms such as GMLVQ (74) achieved near-perfect metabolomics discrimination, and Transformer-based architectures (68) captured temporal EHR dynamics inaccessible to cross-sectional models. This modality-algorithm alignment principle has direct implications for future model development: rather than benchmarking all data types against the same architecture, study designs should prioritize fit-for-purpose methodological choices that reflect the biological question being asked.

Another notable strength across the reviewed literature is the increasing emphasis on validation. More than 60% of included studies incorporated internal cross-validation or external replication cohorts (67–70, 73, 74), reflecting a positive shift towards methodological rigor. The inclusion of diverse population cohorts further enhances generalizability and addresses population specific risk profiles (95). Given documented heterogeneity in NAFLD-HCC risk alleles and clinical presentation across diverse populations (25, 60), such diversity is critical for developing transferable and equitable risk stratification frameworks.

Despite these advances and strengths, several important limitations persist. Small sample-sized and limited data harmonization remains recurring challenges particularly in omics-based and multimodal studies and may compromise model robustness and equity. For instance, Sydor et al. (75) analyzed fecal metaproteomics in a cohort of fewer than 100 patients, while Lewinska et al. (73) investigated lipidomic signatures in 249 participants. While their findings are promising, these relatively small cohorts undermine statistical power, limit stratification analyses, and increase AI/ML systems’ bias. Additionally, many studies relied on single-centre recruitment (69, 72), raising concerns regarding representativeness, transferability, and generalizability and external validity.

A further major limitation to clinical adoption is limited model interpretability. While DL approaches, including those by Xiao et al. (70) and Drozdov et al. (68), often achieve high predictive performance, they typically provide very little insight into mechanistic foundations. The absence of explainability tools such as SHAP or LIME in most studies constrains clinical trust and uptake, particularly for high-stakes decisions such as HCC surveillance (96). Moreover, inconsistent handling of missing data and unclear imputation strategies were frequently observed, further limiting reproducibility and transparency.

**Figure 6:**
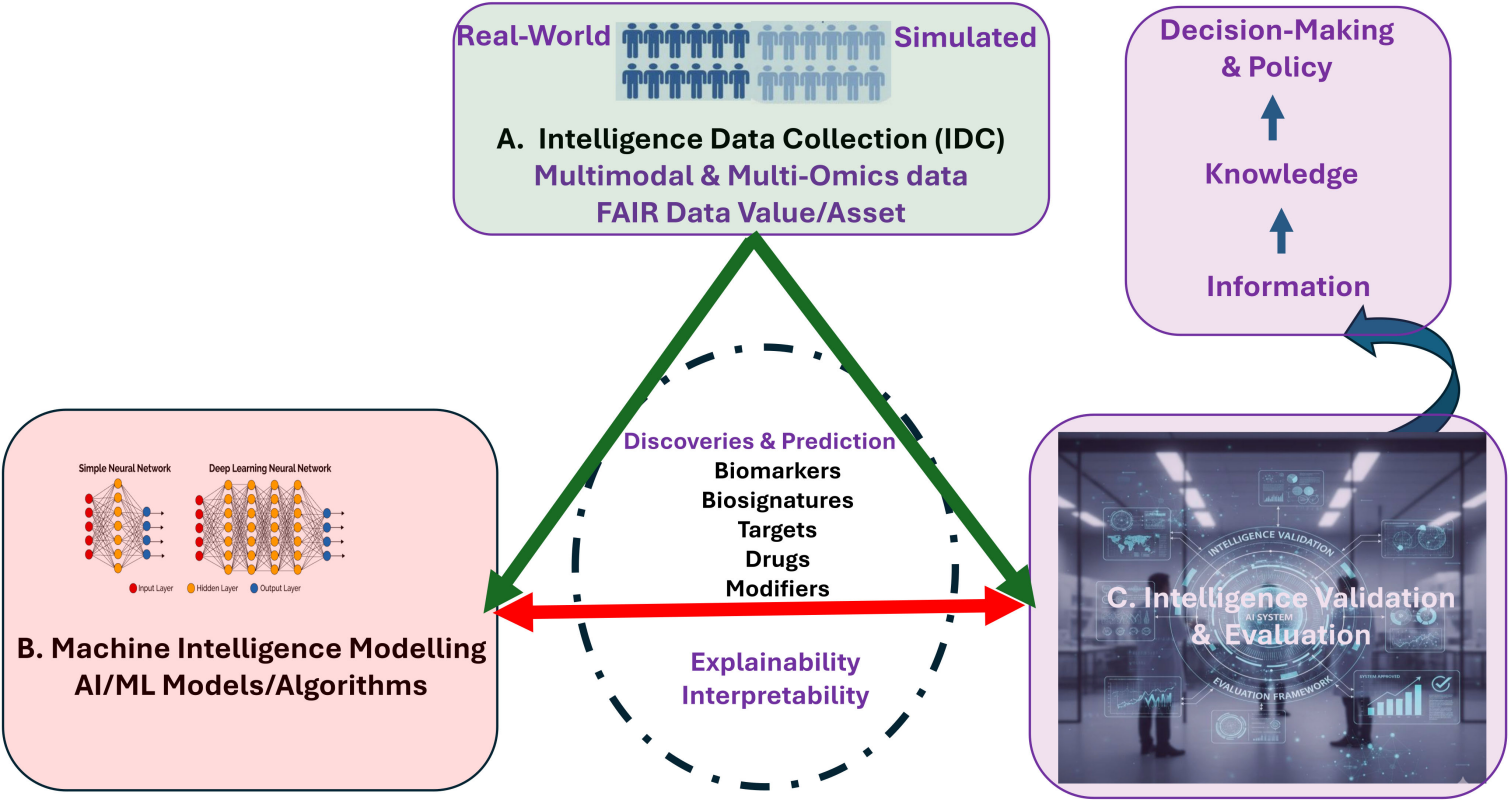
Illustration of integrative components of an AI/ML system to enhance equitability and explained; outcomes through addressing sources of bias at different levels within system **(A)** Data input including; clinical, imaging, and omics data; **(B)** Model development using AI/ML algorithms; **(C)** Clinical output; including risk prediction, early detection, and decision support.

Overall, the risk of selection bias from AI/ML systems has emerged as a recurrent theme, suggesting the need to co-develop ethical, transparent and interpretable AI/ML systems as illustrated in **Figure 6** in addressing source of bias at different levels with the system for equitable and explained outcomes. Taken together, the reviewed studies provide compelling proof-of-concept evidence that AI/ML approaches can outperform conventional methods in MASLD detection, fibrosis assessment, and HCC risk stratification. However, methodological constraints including limited sample sizes, incomplete validation, poor interpretability, explainability and insufficient integration of genomic data currently prevent widespread clinical implementation. Addressing these challenges will require multi-centres that deploy transparent and explainable AI systems within large-scale multimodal data. Europe and some parts of Asia such as China and India offer a strong foundation for advancing AI/ML systems in MASLD detection, fibrosis assessment, and HCC risk stratification. In Europe, large-scale biobanks such as the UK Biobank, FinnGen and Chinese Liver Cancer Atlas provide extensive genomic, clinical, and lifestyle data with long-term follow-up, while national electronic health record (EHR) systems in countries like Denmark, Sweden and China offer rich longitudinal datasets well-suited to AI/ML systems’ applications (97). Parallelly, Europe’s tradition of multi-centre collaboration which is exemplified by initiatives such as the European NAFLD Registry (98), creates the infrastructure needed to validate models across diverse populations and healthcare systems. Incorporating AI/ML systems within such collaborative frameworks could accelerate AI/ML systems’ validation and facilitate their integration into clinical guidelines.

Nonetheless, important limitations remain: many studies relied on relatively small or single-centre datasets, limiting external validity and increasing bias risk, and variability in data harmonization and handling of missing data was frequently reported (72, 73, 75). Limited model interpretability further hampers clinical adoption; while complex models often achieve high predictive accuracy, deep learning approaches provide little insight into underlying mechanisms, potentially reducing clinician trust (96). Regulatory approval is also slowed by the lack of harmonized datasets, standardized pipelines, and validation protocols, although initiatives such as the European Health Data Space (EHDS) could facilitate progress if liver-specific variables are incorporated. Data privacy and governance, especially under the GDPR, complicate cross-border data sharing and the development of multi-centre models; approaches such as federated learning offer potential solutions by enabling model training on distributed datasets without exposing raw patient data (99).

Finally, the clinical utility and cost-effectiveness of AI/ML and PRS-based models remain insufficiently established. Prospective validation and health economic evaluations are essential to determine whether these approaches offer meaningful advantages over current molecular or clinical strategies. Emerging cost-effectiveness analyses in specific contexts, such as AI-assisted liver lesion detection in cirrhotic patients, have begun to demonstrate favourable economic profiles (100). Despite these barriers, the evidence supports the growing role of AI-driven approaches in personalised risk prediction and early detection of MASLD/NAFLD-associated HCC, provided that future research prioritizes transparency, methodological rigor, multimodal and genomic integration, and alignment with regulatory and healthacare frameworks.

## 5 Concluding remarks

This scoping reviews the rapid evolution of AI/ML and PRS methodologies for MASLD detection, fibrosis assessment, and HCC risk stratification, demonstrating that multimodal and omics-integrated models consistently achieve strong predictive performance and offer meaningful advances over conventional approaches. Despite this progress, translation into routine clinical practice depends on resolving four interconnected gaps identified across the reviewed literature: the separation of genomic and AI/ML modelling pipelines, insufficient external validation, population-level representativeness, and the absence of built-in explainability. Addressing these gaps will require PRS-augmented multimodal frameworks that combine established susceptibility variants with longitudinal phenotypic data, subject to prospective validation across diverse healthcare systems. Future efforts should prioritize harmonized data standards, explainable AI techniques, and engagement with regulatory and data governance frameworks, including federated approaches for cross-border model development. With these foundations in place, integrated PRS-AI strategies have the potential to enable personalized HCC surveillance, improve early detection, and ultimately reduce the disease burden of NAFLD on the population scale.

## Supplementary material

Supplementary Table 1 Structured summary of included studies: population, country of study, sample size, disease stage, AI/ML algorithms applied, data modalities, measured outcomes, and reported limitations for all 34 studies included in this scoping review.
