# Supplementary material for: Harnessing AI-driven approaches for detecting metabolic dysfunction-associated steatotic liver disease, assessing fibrosis, and stratifying hepatocellular carcinoma risk: a scoping review
Source: Front Oncol. 2026 Jul 1;16:1803957. doi: 10.3389/fonc.2026.1803957 (PMC13368553; doi:10.3389/fonc.2026.1803957)
Supplement: Supplementary Table 1 — Structured summary of included studies: population, country of study, sample size, disease stage, AI/ML algorithms applied, data modalities, measured outcomes, and reported limitations for all 34 studies included in this scoping review. [file Table1.docx]

***Table 1. Summary of all studies included in the analysis***

| Serial No. | Study (Author, Year) | Study Design | Sample Size | Primary Aim | Machine Learning Methods | Measured Outcome | Main Clinical Focus |
| --- | --- | --- | --- | --- | --- | --- | --- |
| 1. | *Zhang Z, Wang S et al., (2023)* | Retrospective bioinformatics + ML | GSE89632: 64 liver (24 controls, 19 NASH, 21 steatosis); GSE135251: 206 blood (51 controls, 155 NAFLD) | Identify potential genes for NAFLD diagnosis | LASSO, SVM-RFE, Random Forest | AUCs 0.94 (train), 0.91 (validation) | Full Spectrum of MASLD Identification. |
| 2. | *Peng HY, Duan SJ et. al., (2023)* | Retrospective cohort | 709 (578 train; 131 test) | Develop ML models for NAFLD diagnosis | Logistic Regression, RF, XGBoost, GBM, SVM | Best AUC: XGBoost 0.938 | MASLD detection & Fibrosis Assessment |
| 3. | *Razmpour et al., (2023)* | Cross-sectional (Iran) | 513 participants (≥13 yrs) | Predict NAFLD using anthropometry/body composition | kNN, SVM, RBF-SVM, Gaussian Process, RF, NN, AdaBoost, Naïve Bayes | RF accuracy: 82% (fatty liver), 52% (steatosis stage), 57% (fibrosis stage) | MASLD Detection |
| 4. | *Cao Y, Du Y, Jia W, et al. (2023)* | Retrospective bioinformatics + ML (Chinese cohort) | GSE104954 (53 biopsy, 8 controls), GSE89632 (64 liver) | Identify CKD biomarkers in NAFLD patients | ML models with DEGs, PPI networks | AUC: 0.889–1.000 | MASLD Detection |
| 5. | *Ouyang, G., Wu, Z., et al. (2023)* | Bioinformatics + ML with qRT-PCR) | GEO datasets: 115 NAFLD, 106 controls; validation: GSE135251 | Identify cuproptosis-related diagnostic signature for NAFLD | LASSO, RF, SVM-RFE | Diagnostic model with high AUC (train + validation) | MASLD Detection |
| 6. | *Qin, S., Hou, X., et al. (2023)* | Large retrospective screening study (China) | 14,439 adults | Develop ML classifiers for non-invasive NAFLD screening | Decision Tree, RF, SVM, XGBoost | SVM: accuracy 0.801, AUROC 0.850; RF AUROC 0.852 | MASLD Detection |
| 7. | *Verma, N., Duseja, A., et al., (2024)* | Multicenter prospective cohort (14 centers, 8 countries) | 1,656 biopsy-confirmed MASLD patients | Predict significant fibrosis (≥F2) in MASLD | Optimized Random Forest | NPV 0.947, F1 0.754 (overall) | Significant Fibrosis assessment |
| 8. | *Anushiravani et al., (2023)* | Multicenter validation (biopsy-proven MAFLD) | 2,472 patients (674 Egypt; 1,798 other centers) | Validate FIB-6 ML score for cirrhosis/fibrosis | Random Forest (FIB-6 model) | NPV up to 95.8% for cirrhosis; high sensitivity for fibrosis | Significant Cirrhosis assessment |
| 9. | *Stefanakis et al., (2025)* | Multicenter biopsy-confirmed study (Italy, Greece, Australia) | 443 patients (296 Italy, 147 others) | Detect MASH with fibrosis (F2–F3) | Gradient Boosting | AUROC 0.91; Sensitivity 85.3%, Specificity 97% | MASLD Detection |
| 10. | *Yang, B., Lu, H., and Ran,Y., (2024)* | Retrospective + external validation (Dryad + NHANES) | 14,913 total (Dryad + NHANES 2017–2020) | Develop interpretable ML models for NAFLD prediction | Logistic Regression, RF, SVM, DT, KNN, XGBoost, LightGBM (SHAP interpretability) | LightGBM: AUC 0.90 (train), 0.81 (external), Accuracy 87.2%, F1=0.92 | MASLD Detection |
| 11. | *Alizargar, A., Chang, Y., et al., (2024)* | Cross-sectional ML with gender stratification (U.S.) | 2,505 participants | Develop ensemble ML for cost-effective NAFLD detection | RF, KNN, Logistic Regression, SVM, XGBoost, DT, Ensemble | Ensemble: Accuracy ~0.99, AUC ~1.00 | MASLD Detection |
| 12. | *Cao et al., (2024)* | Longitudinal cohort (Beijing) | 22,140 participants | \|  \| \| --- \|  \| Build predictive ML model for NAFLD risk \| \| --- \| | LASSO feature selection; XGBoost, others tested | XGBoost: Accuracy 0.835, AUC 0.914 | MASLD Detection |
| 13. | *Qin,J., Cao, P., et al., (2023)* | Multi-dataset + bioinformatics + ML + functional assays | 282 samples (human, mouse, scRNA-seq; 5 GEO datasets) | Identify ferroptosis-related gene biomarkers for NAFLD | LASSO, SVM, RF, Boruta; consensus clustering | ANXA2 identified as key biomarker; functional validation in HepG2 | MASLD Detection |
| 14. | *Zhang et al., (2024)* | Cross-sectional (China) | 978 examined, 916 with TE data | Develop low-cost NAFLD prediction models | Logistic Regression, KNN, SVM, RF, LightGBM, XGBoost | RF: AUROC ~0.91; simplified RF model AUROC ~0.88 | MASLD Detection |
| 15. | *Tian, F., Zhao, Y., et al. (2025)* | Bioinformatics + ML (human + mouse validation) | 214 humans (132 NAFLD, 82 controls) + 6 mouse samples | Explore PANoptosis-related genes in NAFLD diagnosis/subtyping | ML model + SHAP interpretability | AUROC ~0.976; subtype classification via hub genes | MASLD Detection |
| 16. | *Chen et al., (2025)* | Multimodal ML (DeepFLD, >6,000 participants) | >6,000 participants | Predict NAFLD using multimodal data (clinical, imaging, facial photos) | DeepFLD (multimodal deep learning) | Outperformed clinical-only models; maintained accuracy with only facial images | MASLD Detection |
| 17. | *Masaebi et al., (2024)* | Rural Iranian cohort (Fasa study) | 6,180 adults | Predict MASLD with body comp + lab data | Logistic Regression, Naïve Bayes, SVM, LightGBM | Logistic Regression: Accuracy 88%, AUC 0.92 | MASLD Detection |
| 18. | *Lim et al., (2024)* | Longitudinal, survival ML (Asian cohorts) | 25,599 Korean (train); 16,173 Chinese (validation) | Predict NAFLD onset in general population | Random Survival Forest, Extra Survival Trees | Accuracy ~0.75 in Chinese external validation | MASLD Detection |
| 19. | *Shen, C. H., Huang, R., et al. (2025)* | Cross-sectional, Taiwan MJ cohort | 1,501 | Detect NAFLD using VOCs + clinical data | RF, 9 others | Accuracy ↑ from 72.2% to 77% with VOCs | MASLD Detection |
| 20. | *Bao et al., (2025)* | Hospital-based, Chinese T2DM cohort | 1,553 | Predict lean NAFLD in T2DM | RF, LR, Boruta, LASSO | RF accuracy 0.789; key predictors identified | MASLD Detection |
| 21. | *An et al., (2025)* | Cross-sectional, single-centre | 536 | Evaluate LBT (H₂, CH₄) for NAFLD prediction | XGBoost, RF, SVM, KNN, LR | XGBoost AUROC ~0.88; LBT improved sensitivity | MASLD Detection |
| 22. | *Fang, Z., Liu, C., et al. (2024)* | ML + scRNA-seq analysis | 176 (104 NAFLD, 72 controls) + 80 validation | Identify NET-related biomarkers in NAFLD | LASSO, SVM-RFE, RF | Hub genes ZFP36L2, PHLDA1 predictive; immune-metabolic link | MASLD Detection |
| 23. | *Jamialahmadi et al., (2025)* | Clinical, bariatric surgery cohort | 512 | Predict liver fibrosis in obesity | LR, SVM, NN, NB | LR AUC 0.73; SVM sensitivity 83% | Significant Fibrosis Assessment |
| 24. | *Makkena & Natarajan, (2025)* | Retrospective dataset (Kaggle) | 418 (274 non-cirrhotic, 144 cirrhotic) | Enhance cirrhosis diagnosis with XAI | XGBoost, RF, LR, NB, ensembles | XGBoost accuracy 91.2%; SHAP for interpretability | Significant Cirrhosis Assessment |
| 25. | *Wang et al., (2024)* | Bioinformatics + multi-cohort validation | GEO + 320 NAFLD, 300 controls, 870 liver disease pts | Develop diagnostic gene model for NAFLD | LASSO, SVM-RFE | 4-gene model AUC 0.997 | MASLD Detection |
| 26. | Zhang *et al*., (2024) | Bioinformatics + ML | GEO RNA-seq datasets | Build prognostic 6-gene signature for NAFLD | SVM, RF, LASSO, XGBoost | SVM best; 6-gene nomogram predictive | MASLD Detection |
| 27. | Xiao *et al*. | Observational, prognostic modelling | 36,490 | Early detection & prognosis stratification of NAFLD | Transformer neural networks, Random Forest, XGBoost | AUROC for NAFLD diagnosis & prognosis | MASLD Detection |
| 28. | Sydor *et al*. | Diagnostic biomarker study | 80 | Identify fecal metaproteomic biomarkers for NASH ± HCC | Random Forest, Gradient Boosting | Classification accuracy, AUROC | MASLD Detection |
| 29. | Qin *et al*. | Cross-sectional screening study | 14,439 | Screen NAFLD using ML classifiers | Support Vector Machine, Random Forest | AUROC for NAFLD screening | MASLD Detection & Hepatocellular carcinoma outcomes |
| 30. | Moolla *et al*. | Diagnostic accuracy study | 275 | Non-invasive NAFLD staging using urinary steroid metabolome | GMLVQ, Logistic Regression | AUROC for fibrosis/cirrhosis detection | MASLD Detection |
| 31. | Lewinska *et al*. | Diagnostic biomarker study | 249 | Lipidomic profiling for NAFLD-associated HCC | Random Forest, Gradient Boosting | AUROC for HCC detection | MASLD Detection & Hepatocellular Carcinoma outcomes |
| 32. | Ginter-Matuszewska *et al*. | Diagnostic accuracy study | 484 | Early-stage fibrosis & steatosis detection | Random Forest, SVM, KNN, Decision Tree, MLP, Naive Bayes | AUROC for fibrosis & steatosis detection | Fibrosis assessment |
| 33. | Drozdov *et al*. | Observational prognostic study | 940 | Predict all-cause mortality in MASLD | Transformer neural networks, Cox regression | AUROC for mortality prediction | MASLD Detection |
| 34. | Atabaki-Pasdar *et al*. | Observational, GWAS-based | 3029 | Predict fatty liver & explore etiology | Random Forest, Gradient Boosting, Logistic Regression | AUROC for fatty liver prediction | MASLD Detection. |
